# Reasons for non-vaccination against HPV and future vaccination intentions among 19-26 year-old women

**DOI:** 10.1186/1472-6874-10-27

**Published:** 2010-09-01

**Authors:** Gregory D Zimet, Thomas W Weiss, Susan L Rosenthal, Margaret B Good, Michelle D Vichnin

**Affiliations:** 1 Department of Pediatrics, Indiana University School of Medicine, 410 W. 10th Street, HS 1001, Indianapolis, IN, 46202, USA; 2 Merck & Co., Inc. West Point, PA, USA; 3 Department of Pediatrics, Columbia University, New York, NY, USA; 4 I3 Innovus, Eden Prairie, MN, USA

## Abstract

**Background:**

Despite CDC recommendations regarding universal catch-up vaccination against human papillomavirus (HPV), only about ten percent of young adult women in the United States have been vaccinated. The purpose of this study was to better understand reasons for non-vaccination among insured 19-26 year-old women and to evaluate future vaccination intentions.

**Methods:**

We used an administrative claims database from a large US managed care plan to identify women aged 19-26 for receipt of a mailed survey. From a sample of 1,375 women with no evidence of HPV vaccination from June 1, 2006 through April 30, 2007, 222 completed surveys were received, of which 185 were eligible for this analysis. The main outcome measures were unvaccinated women's attitudes and vaccine awareness, likelihood of future action regarding the vaccine, and reasons for inaction.

**Results:**

Among the 185 non-vaccinees, 25.4% were married, 83.2% were white, and 89.2% had a college or higher level education. The vaccine was described as very important by 32.4% of subjects, and 30.1% had discussed the vaccine with a doctor and received a doctor's recommendation. Half or fewer of respondents were "very" or "extremely" likely to discuss the vaccine with their doctor (50.0%), do additional research on the vaccine (42.6%), ask a doctor to get the vaccine (37.5%), or make an appointment to get the vaccine (27.8%), while 48.0% were "somewhat", "very", or "extremely" likely to do nothing to get the vaccine. Among the latter, reasons for taking no action included being married or in a monogamous relationship (54.9%), belief that the vaccine is too new (35.4%), not having enough information about the vaccine (31.7%), concerns about side effects (24.4%), and uncertainty about insurance coverage (24.4%).

**Conclusions:**

Educational interventions may be needed to enhance HPV vaccination rates among 19-26 year-old women, particularly regarding information about vaccine safety, vaccine efficacy, insurance coverage, and the value of vaccination to women in monogamous relationships.

## Background

Human papillomavirus (HPV) is the most common sexually transmitted infection in the United States [1]. Low-risk HPV types 6 and 11 cause 90% of cases of genital warts, while high-risk HPV types 16 and 18 are responsible for 70% of cervical cancers and also cause cancers of the anus, vagina, vulva, and head and neck [2,3].

Two vaccines have been developed for the prevention of HPV infection. One is bivalent, targeting high-risk HPV types 16 and 18 [4], and approved by the FDA in October, 2009 for the prevention of cervical cancer and certain precancerous lesions caused by HPV infection [5]. The quadrivalent vaccine, which targets HPV types 6, 11, 16, and 18 [6,7], was approved by the FDA in 2006 for the prevention of cervical cancer, certain precancerous lesions, and genital warts in females and, in 2009, for the prevention of genital warts in males [8,9]. In 2007 the CDC's Advisory Committee on Immunization Practices (ACIP) recommended routine vaccination of 11-12 year-old females with three doses of the quadrivalent vaccine and catch-up vaccination of 13-26 year-old females who have not been vaccinated previously [10]. Recent, provisional recommendations from the ACIP extend these recommendations to the bivalent vaccine and also state that the quadrivalent HPV vaccine may be given to males aged 9 through 26 years to reduce their likelihood of acquiring genital warts [11].

Despite the ACIP recommendation, vaccination coverage rates remain low (≤36%) [12,13], particularly among 18-26 year-old females (10-12%) [14,15]. Given that there is demonstrated benefit to immunizing young adults, even if they are already sexually active [16], it is important to gain a better understanding of the patient factors that are associated with vaccination and non-vaccination in this age group.

A previous article by the authors using the same database as the present study compared vaccinated with non-vaccinated women and demonstrated the importance of a physician's recommendation to vaccination decisions [17]. The present analyses were conducted to explore in greater depth the reasons for limited uptake of the HPV vaccine among young adult women who have yet to be vaccinated.

## Methods

### Study design

Subjects were identified using medical and pharmacy claims from a large US managed care plan affiliated with i3 Innovus. The health plan provides fully insured coverage of physician, hospital, and prescription drug services to more than 15 million patients across the four major geographic regions of the United States: the Northeast, Midwest, South, and West. A self-administered survey assessed factors related to an individual's decision to receive or not receive HPV vaccine administration. One of the authors (MBG) is an employee of i3 Innovus and accessed the claims data to identify patients who qualified for this study. This research was carried out in compliance with the Declaration of Helsinki (http://www.wma.net/en/30publications/10policies/b3/17c.pdf) and ethics approval for the study was obtained by the Copernicus Group Independent Review Board (http://www.copernicusgroup.com).

### Patient sample

Women were identified in the health plan database if: they received a new claim for the HPV vaccine or any outpatient medical claim during an identification period defined as January 1 to April 30, 2007; were 19-26 years of age on the index date (i.e., first date of claim during identification period); and were continuously enrolled in the health plan for 6 months prior to and 12 months following the index date. Women were excluded if they had a medical claim related to pregnancy, delivery, or cervical cancer during the identification period. A total of 611,955 women met the above criteria.

Two samples of 1,375 women were randomly selected from among women who had: (1) a first HPV vaccine dose during the identification period, and (2) no doses of HPV vaccine during the identification period or during the 6 months before that period [17]. The second group (non-vaccinees) constitutes the patient sample for this analysis. The sample size of 1,375 women per group (vaccinated, unvaccinated) was chosen to ensure that the final sample sizes provided adequate power to detect differences between the two groups for the analyses reported in the previous publication [17].

### Survey administration

Survey packets were mailed beginning in April 2008. Packets contained an invitation letter, an informed consent form, a 7-page survey assessing factors related to an individual's decision to receive or not receive the HPV vaccine, and a payment of $10 as compensation for participation. Reminder post-cards were sent one week following the initial mailing, and a second survey packet was sent to non-respondents after three weeks. The total survey collection period was 8 weeks.

### Survey content

The survey collected standard demographic and health status information (Additional file 1: HPV vaccine questionnaire 18-26). Questions addressed attitudes toward reproductive health, vaccine awareness, and discussion of the vaccine with a physician. Non-vaccinees were asked about the likelihood of receiving the vaccine in the future.

To assess attitudes toward reproductive health respondents were asked to rate their level of agreement, from 1 (strongly disagree) to 6 (strongly agree), with statements such as "Cervical cancer is a devastating disease," "Genital warts are an embarrassing condition," and "I am comfortable discussing sexual health issues with a doctor or nurse."

To assess vaccine awareness, respondents were first asked if they had ever heard of a vaccine to help prevent cervical cancer or HPV infection. If yes, they were then asked a series of questions about their perception of the personal importance of the HPV vaccine by the question, "How important do you think the vaccine to help prevent cervical cancer is for you?" Possible answers were 'not at all important', 'not very important', 'somewhat important', and 'very important'.

The questions that addressed discussion with and recommendation by a physician were: "Have you discussed the vaccine to help prevent cervical cancer with a doctor?" and "Did a doctor recommend that you get the vaccine to help prevent cervical cancer?" If the respondent reported that a doctor did recommend the vaccine, she was asked, "How strongly did the doctor recommend you get the cervical cancer vaccine on a scale from 1 to 5?" A rating of 1 corresponded to the response that 'a doctor did not strongly recommend the vaccine' and a rating of 5 meant that 'a doctor strongly recommended the vaccine'.

The likelihood of taking additional action regarding the HPV vaccine was assessed via the question, "If you have not received a single dose of the cervical cancer vaccine, in the future how likely are you to" followed by 5 choices: 'Ask a doctor to get this vaccine?', 'Do additional research on this vaccine?', 'Discuss the vaccine with a doctor?', 'Make an appointment to get the vaccine?', and 'Do nothing to get the vaccine?'. The answers were measured on a 5-point Likert-type scale, ranging from 'not at all likely' (1) to 'extremely likely' (5).

Respondents who were somewhat, very, or extremely likely (3-5 on the Likert-type scale) to 'do nothing to get the vaccine' were asked their reasons for inaction. Possible answers were 'I am not sexually active', 'The vaccine is too new', 'I am married or in an exclusive (monogamous) relationship', 'I am unsure if my insurance would cover the vaccine cost', 'I do not have enough information about this vaccine', 'I am concerned about the side effects', 'I am pregnant or trying to conceive', 'My doctor recommended against getting the vaccine', and 'I cannot afford the cost of the vaccine'. Respondents were instructed to 'check all that apply' and were given the option of articulating their own reason(s).

### Data analysis

A descriptive analysis was conducted to determine the attitudes of non-vaccinees toward the HPV vaccine, their awareness of the vaccine, their interaction with a physician relating to the vaccine, the likelihood of their taking future action regarding HPV vaccination, and their reasons for inaction.

## Results

### Patient sample

Of the 1,375 surveys sent out to non-vaccinees, 222 were returned complete, for a response rate of 16.1%. Thirty-seven non-vaccinees were subsequently determined to be ineligible due to disenrollment or vaccination after the identification period, leaving 185 surveys eligible for analysis. As reported in our earlier paper, there were no differences between respondents and non-respondents with respect to region of residence or number of comorbidities [17]. Respondents were statistically significantly younger (mean age 21.6) than non-respondents (mean age 22) but this was not a clinically meaningful difference.

The sociodemographic characteristics and health status of the respondents are shown in Table 1. The mean age (SD) was 22.4 (2.3) years. The majority were not married (74.6%) and had good, very good, or excellent health (92.4%). Most were white (83.2%) and had a college or higher level education (89.2%).

**Table 1:** Respondent characteristics*

	N	%
Marital Status		
Not married	138	74.6
Married	47	25.4
Overall health status		
Poor/Fair	14	7.7
Good	50	27.3
Very good	77	42.1
Excellent	42	23.0
Education		
High school or less	20	10.8
Some college, or but no degree	82	44.3
Two year degree or college graduate	66	35.7
Graduate school	17	9.2
Race/Ethnicity		
White, non-Hispanic	154	83.2
Black, non-Hispanic	5	2.7
Hispanic	14	7.6
Other	12	6.5
Employment status		
Full-time	96	51.9
Student status		
Student	56	30.3

* Mean age (SD): 22.4 (2.3) years; *N = 185

### Attitudes, vaccine awareness, and physician interaction

Most respondents agreed that cervical cancer is a devastating disease (93.5%) and that genital warts are an embarrassing condition (90.0%), and most reported being comfortable discussing sexual health issues with a doctor or nurse (73.0%; Table 2). Of the 185 respondents, 176 reported ever hearing of a vaccine to help prevent cervical cancer or HPV infection. Of these, less than one third (32.4%) thought that the HPV vaccine was very important for them. Likewise, 53 (30.1%) reported discussing the HPV vaccine with their doctor and receiving their doctor's recommendation for the vaccine. Only 15.3% of all respondents received a 'strong' recommendation, while of the 53 women that received a doctor's recommendation, 27 (50.9%) received a strong recommendation.

**Table 2:** Attitudes, vaccine awareness, and physician interaction

	Responses	N	%
Cervical cancer is a devastating disease (% agreement†)	185	173	93.5
Genital warts are an embarrassing condition (% agreement†)	180	162	90.0
I am comfortable discussing sexual health issues with a doctor or nurse (% agreement†)	185	135	73.0
Vaccine to prevent cervical cancer was very important to them ‡	176	57	32.4
Doctor discussed and recommended vaccine to help prevent cervical cancer	176	53	30.1
Received a strong recommendation§ from doctor to get cervical cancer vaccine	176	27	15.3

†Strongly or moderately agree (5 or 6 on the 6-point Likert scale).

‡Checking "very important" to a question stated as, "How important do you think the vaccine to prevent cervical cancer is for you?" with possible answers of not at all, not very, somewhat, or very important.

§Equivalent to a score of 4 or 5 on the 5-point Likert scale.

### Likelihood of taking additional action

Most respondents reported being at least somewhat likely to discuss the HPV vaccine with a doctor in the future (84.7%), to do additional research on the vaccine (75.0%), ask a doctor to vaccinate them (69.9%), or to make an appointment to be vaccinated (60.2%; Table 3). Substantially fewer patients were very or extremely likely to carry out these actions (Table 3).

**Table 3:** Likelihood of taking additional action regarding HPV vaccination

	Responses	Somewhat, very, or extremely likely		Very or extremely likely*	
		N	%	N	%
Discuss the vaccine with a doctor	176	149	84.7	88	50.0
Do additional research on vaccine	176	132	75.0	75	42.6
Ask a doctor to get vaccine	176	123	69.9	66	37.5
Make an appointment to get the vaccine	176	106	60.2	49	27.8
Do nothing to get the vaccine	175	84	48.0	37	21.1

* 'Somewhat likely' response category removed.

### Reasons for taking no additional action

The reason most commonly given for taking no additional action regarding HPV vaccination was being married or in a monogamous relationship (54.9%; Table 4). About a third of the respondents thought the vaccine was too new (35.4%) or that they did not have enough information about it (31.7%). One fourth (24.4%) were concerned about the vaccine's side effects. Some were unsure of their insurance coverage (24.4%), and a few were concerned about the cost of the vaccine (14.6%).

**Table 4:** Reasons for taking no additional action regarding HPV vaccination*

	N	%
Married or in an exclusive (monogamous) relationship	45	54.9
Vaccine is too new	29	35.4
Do not have enough information about vaccine	26	31.7
Concerned about side effects	20	24.4
Unsure of insurance coverage	20	24.4
Cannot afford cost of vaccine	12	14.6
Pregnant or trying to conceive	9	11.0
Not sexually active	8	9.8
Doctor recommended against getting vaccine	4	4.9

*Among those stating that they were somewhat likely, very likely, or extremely likely (3-5 on a 5-point scale) to do nothing to get the vaccine (N = 82). Two respondents had missing data. Based on "all that apply" responses.

## Discussion

The implications of this study must be considered in the context of several limitations. The opinions recorded in this survey, for example, are not necessarily representative of those of the entire target population, because of a potential response bias towards non-compliant patients. The response rate of 16.1% was rather low and was lower than that of the vaccinated subgroup (28%) [17]. In addition, more than 80% of respondents were white and all study subjects had access to health care and were insured. The results regarding intent to get vaccinated, while interesting, may not ultimately reflect future behavior. For instance, a meta-analytic review of Theory of Planned Behavior research reported a relative modest average correlation of 0.47 between intentions and subsequent health behaviors [18].

Although the majority of unvaccinated respondents in this study recognized the potential impact of cervical cancer and genital warts, only about one in three considered vaccination against HPV to be important to them. Half of respondents reported being very or extremely likely to discuss the vaccine with a doctor but almost half (48.1%) were at least somewhat likely to do nothing to pursue vaccination. The most frequent reason for inaction was being married or in a monogamous relationship, but other reasons relating to uncertainty about the vaccine and insurance/cost issues were common.

These reasons for inaction echo findings of previous studies, which noted a perceived lack of need, concerns about the vaccine, and perceived barriers as factors in the decision not to be vaccinated. A perceived lack of need, usually due to sexual inactivity or low risk, was found to be a factor in previous population surveys of this age group [14,15]. Safety of the vaccine was also raised as an issue in these surveys [14,15]. In a study of college-age women, those who had decided to forego vaccination were less sure of the vaccine's safety and were less knowledgeable about risk factors, transmission, and methods of detection of HPV than were women who had been vaccinated [19].

As in the present study, a relatively small proportion of women (7%) in the National Immunization Survey (NIS) 2007 were concerned about cost or insurance issues [15]. Cost was also a barrier reported by college women who had chosen not to receive the vaccine [19]. However, larger numbers reported other barriers to vaccination, including lack of time or failure to make a doctor's appointment (21% in the NIS 2007) [15]. Similarly, in a follow-up study that included women aged 13-26, 45% of young women who had not yet been vaccinated had simply not returned to the clinic in the six months since the baseline study [13].

In our previous paper published from this data set we found that those respondents who reported physician discussion/recommendation of HPV vaccination were significantly more likely to be in the vaccinated group [17]. The women described here remained unvaccinated, and only 30.1% had received a recommendation from their physician to be vaccinated, with only 15% receiving a strong recommendation.

Responses to questions about taking additional action suggested ambivalence on the part of non-vaccinees (almost 70% were at least somewhat likely to ask a doctor to get the vaccine, while 48% expressed being at least somewhat likely to do nothing). This inconsistency may have resulted from the choice of cutoff ("somewhat," "very," and "extremely" likely) in the accepted responses. There was no discrepancy if the threshold was set at "very" or "extremely" likely, when from 27.8% to 50.0% would take some action regarding the vaccine, and 27.1% would do nothing. Ambivalence about vaccination is, however, consistent with the lack of correlation between the intention to be vaccinated and actual vaccination observed in one post-vaccine study [13]. This result may explain the discordance in the United States between the findings of studies carried out before the vaccine became available, which showed favorable attitudes toward HPV vaccination (74-89% acceptance rate) [20-23], and current low vaccination rates (5-36%) [12,13]. Alternatively, these low rates of vaccination may reflect an inefficient method of vaccine delivery for young adult women in the United States. In countries with school-based vaccination programs for girls, there is good agreement between rates of pre-vaccine acceptance and actual vaccination. In England, for instance, more than 80% of people surveyed were in favor of HPV vaccination [24], and school-based programs have reported more than 70% uptake of the first vaccine dose among 12-13 year-olds [25,26]. In Australia, the pre-vaccine acceptance rate was about 80% [27]. When a school-based program in Queensland contracted with a general practice to provide recommended immunizations, uptake of all three doses of the HPV vaccine was 79% [28].

## Conclusions

These findings suggest that educational interventions may be necessary to enhance HPV vaccination rates among young adult women. Although some of these women currently may be at low risk for infection, using assessment of risk as a basis for deciding who to vaccinate is unlikely to be an effective approach [29]. Further, most young women have not been infected with all vaccine-related HPV types and therefore can still achieve some degree of protection through vaccination [16]. The most effective way to protect the greatest number of women against HPV infection is to optimize provision of vaccine to all women eligible for vaccination [29]. Therefore, delivery of information to young women about vaccine safety, vaccine efficacy, insurance coverage, and the value of vaccination to women in monogamous relationships may be needed to better inform those who decline HPV vaccination.

## Competing interests

GDZ and SLR have served as paid research consultants to Merck & Co., Inc. They are also co-principal investigators and co-investigators on two investigator-initiated research studies funded by Merck & Co., Inc.

TWW and MDV are employees of Merck & Co., Inc.

MBG is an employee of I3 Innovus.

The research reported in this article was funded by Merck & Co., Inc.

## Authors' contributions

GDZ took primary responsibility for designing the study and writing the paper and contributed to statistical analyses; TWW contributed to study design, statistical analyses, and writing of the paper; SLR contributed to study design and helped write the paper; MBG was responsible for data acquisition and helped write the paper; MDV contributed to study design and helped write the paper. All authors read and approved the manuscript.

## Pre-publication history

The pre-publication history for this paper can be accessed here:

http://www.biomedcentral.com/1472-6874/10/27/prepub

## Supplementary Material

Additional file 1 **HPV vaccine questionnaire 18-26**. This questionnaire was directed toward 18-26 year old women and assessed HPV vaccination status and reasons for vaccination/non-vaccination as well as knowledge and attitudes about HPV and HPV vaccination. Click here for file
