# Supplementary material for: Reasons for non-vaccination against HPV and future vaccination intentions among 19-26 year-old women
Source: BMC Womens Health. 2010 Sep 1;10:27. doi: 10.1186/1472-6874-10-27 (PMC2941477; doi:10.1186/1472-6874-10-27)
Supplement: Additional file 1 — HPV vaccine questionnaire 18-26. This questionnaire was directed toward 18-26 year old women and assessed HPV vaccination status and reasons for vaccination/non-vaccination as well as knowledge and attitudes about HPV and HPV vaccination. [file 1472-6874-10-27-S1.PDF]

DO NOT USE WITHOUT AUTHORS' PERMISSION

Survey Booklet

Survey A

19-26 Year Old Women

Please return this booklet using the enclosed postage-paid envelope by May 19, 2008.

Thank you for participating in this important study about women's health issues and vaccinations.

Instructions

To participate in the study, please do the following:

1. Read the **Subject Information and Consent Form** on pages 1-4

    → sign and date page 4

2. Read the **Authorization to Use and Disclose Personal Health Information for Research Purposes** and the **Health Information Release Form** on pages 5-6

    → sign and date page 6

3. Read the **Survey Directions** on page 7

    → complete the survey on pages 7-13

4. **Return this completed booklet using the enclosed postage-paid envelope by May 19, 2008.**

The $10 bill that came with the survey is yours to keep. It is to thank you for participating in this study. Thank you for your time and effort to complete this important survey.

Questions About this Study?
If you have any questions about this study or would like to discuss it further, please contact Andrea Nugent, i3 Innovus Project Manager, toll-free at **1-800-486-0364** or e-mail at andrea.nugent@i3innovus.com.

Questions or Concerns About Your Treatment or Medical Products You May Be Taking?
If you have any questions about your treatment, or if you experience any health problems during the study, please contact your doctor directly.

If you are experiencing reactions or problems with any medications you are taking please contact the **U.S. Food and Drug Administration – MedWatch program**. MedWatch is the Food and Drug Administration's (FDA) program for reporting serious reactions and problems with medical products, such as drugs and medical devices.

If you think you or someone in your family has experienced a serious reaction to a medical product, please contact the FDA's toll-free information line, 1-888-INFO-FDA (1-888-463-6332) or access the FDA MedWatch Website at http://www.fda.gov/medwatch/report/consumer/consumer.htm

12125 Technology Drive
Eden Prairie, MN 55344
Tel (952) 833-7777 | Fax (952) 833-6045
www.i3magnifi.com

Copernicus Group IRB

Approved: 03/20/2008

SUBJECT INFORMATION AND CONSENT FORM AND AUTHORIZATION TO USE AND DISCLOSE PERSONAL HEALTH INFORMATION FOR RESEARCH PURPOSES (19-26 years old)

**NAME OF RESEARCH STUDY:**	Factors Affecting Administration and Completion of Human Papillomavirus (HPV) Vaccine Regimens
**PROTOCOL #:**	95163
**SPONSOR:**	Merck & Co., Inc.<br>Whitehouse Station, NJ<br>United States
**PRINCIPAL INVESTIGATOR:**	**Barbara McMorris PhD**
**RESEARCH SITE ADDRESS:**	i3 Innovus<br>12125 Technology Drive<br>Mailroute: MN002-0258<br>Eden Prairie, MN 55344<br>United States
**DAYTIME TELEPHONE NUMBER(S):**	Andrea Nugent MPH, CCRC<br>Project Manager<br>1-800-486-0364 (toll-free)<br><br>Barbara McMorris PhD<br>952-833-7083

Purpose of the Subject Information and Consent Form

This consent form may contain words that you do not understand. Please call the study doctor or other designated study staff to explain any words or information that you do not clearly understand.

The purpose of this consent form is to give you information about the research study and, if signed, will give your permission to take part in the study. You must sign this consent form in order to take part in the study. The consent form describes the purpose, procedures, benefits, and risks of the research study. You should read this consent form carefully. You should take part in this study only if you want to do so. You may withdraw from this study at any time without penalty or loss of benefits to which you are otherwise entitled. Please read this consent form carefully and ask as many questions as needed. You should not sign this consent form if you have any questions that have not been answered to your satisfaction.

Purpose and Description of the Research Study

You are being asked to take part in a research study sponsored by Merck & Co., Inc., and conducted by i3 Innovus, a division of Ingenix Pharmaceutical Services. Ingenix is a subsidiary company of UnitedHealth Group, Incorporated. You are being asked to take part in this study because you are a UnitedHealth Group insurance beneficiary, are female and are between the ages of 19 and 45. You were randomly selected to participate in this survey study from a large list of eligible participants.

The goal of this research study is to learn more about your attitudes about your health and the use of vaccinations. In particular, this survey will ask about your opinions regarding vaccination against cervical cancer. Approximately 800 women in the US ages 19-26 are expected to participate in this survey study.

Study Procedures

You will be asked to complete a survey. The survey asks questions about your understanding of human papillomavirus (HPV), cervical cancer prevention, and cervical cancer vaccination (shot). At the end, you will be asked some general questions about yourself. It should take about 15-20 minutes to complete the survey. You have been provided with a postage-paid envelope for returning the completed survey booklet.

As part of this study, Ingenix may review any submitted health care and pharmacy claims related to this survey study during the study period, and up to 2 years prior to your participation in this study.

Risks or Discomforts

There are no anticipated physical health risks related to participation in this study. Some of your personal health information may be viewed by research staff that may not ordinarily see it, so there is a risk of loss of confidentiality.

Possible Benefits

There is no direct benefit to you from participating in this survey study. However, your participation may help improve future patient care and cervical cancer prevention for other women.

Payment to Subject for Participation

You have received $10.00 cash in advance of completing and returning the survey booklet by mail. You may keep the $10.00 cash payment even if you choose not to participate in this study.

Costs

There are no costs to you associated with your participation in this study.

Alternative Treatments

Your alternative is to not take part in this study.

Confidentiality and Release of Medical Records
We will protect information about you and your taking part in this research study to the best of our ability. If information about this study is published, your name will not be given. However, the U.S. Food and Drug Administration (FDA) and the Copernicus Group Independent Review Board (IRB) may sometimes look at the medical records and study information of those who take part in this study. A court of law could order medical records shown to other people, but that is unlikely. Therefore, absolute confidentiality cannot be guaranteed.

Legal Rights
You do not waive any legal rights by signing this consent form.

Voluntary Participation
Your decision to take part in this research study is completely voluntary. There will not be any penalty or loss of benefits to you if you decide not to take part.

Withdrawal
You may stop your participation in this study at any time. Your decision will not result in any penalty or loss of benefits to which you are entitled. You may stop your participation at any time by informing Andrea Nugent that you wish to withdraw from this study.

Contact for Questions
If you have any questions about this study, contact:

Project Manager Name: Andrea Nugent MPH, CCRC

Daytime telephone number(s): 1-800-486-0364 (toll-free)

Questions regarding your health should be directed to your doctor. If you experience any illness, health problems or concerns, please contact your physician directly.

If you have questions about your rights as a research subject, you may contact Copernicus Group Independent Review Board (IRB) at 1-888-303-2224 (toll-free). An IRB is a group of scientific and non-scientific individuals who perform the initial and ongoing ethical review of the research study with the study subject's safety and welfare in mind. Copernicus Group IRB has reviewed and approved the research study described in this Subject Information and Consent Form. If you have study-related comments, complaints or concerns, you should first contact the study investigator. Please call the IRB if you want to talk to someone other than the study investigator or have difficulty reaching the study investigator. For further information regarding the research process and your role as a research subject, you may visit the Copernicus Group IRB website at www.cgirb.com.

Do not sign this consent form unless you have had a chance to ask questions and have received satisfactory answers to all of your questions.

Subject's Statement of Consent

Factors Affecting Administration and Completion of Human Papillomavirus (HPV) Vaccine Regimens

I have read the above information.

I have had an opportunity to ask questions about this research study and all my questions have been answered to my satisfaction. I have been told what the possible risks and benefits are from taking part in this research study. By signing this consent form, I do not give up my legal rights. I voluntarily agree to take part in this research study.

____
Signature of Subject **Date**

____
Printed Name of Subject

Authorization to Use and Disclose Personal Health Information for Research Purposes

Federal regulations give you certain rights related to your health information. These include the right to know who will be able to get the information and why they may be able to get it. The study investigator must get your authorization (permission) to use or give out any health information such as your name, address, telephone number or other facts that might identify you.

During the study, the investigator or other designated staff will record health information about you (your "records") in order to carry out this study. The U.S. Food and Drug Administration (FDA), Copernicus Group Independent Review Board, i3 Innovus and i3 Innovus' business associates may sometimes look at the study information of those who take part in this study. Your personal health information may further be shared by the groups above. If shared by them, the information will no longer be covered by the Privacy Rule. However, these groups are committed to keeping your health information confidential. This includes information created or collected during the study.

The results of this study will be shared with the study sponsor and its representatives. The results may be presented at meetings or published in scientific journals, but your identity will not be disclosed. However, the information may be reviewed by the study sponsor and Copernicus Group Independent Review Board.

You have the right to see and get a copy of your records related to the study for as long as the study doctor has this information. However, by signing this Authorization you agree that you might not be able to review or receive some of your records related to the study until after the study has been completed.

By signing this form, you allow the study investigator and study staff to use your records to carry out this study. If you refuse to give permission, you will not be able to take part in this research.

All information that has already been collected for study purposes will be sent to the study sponsor.

You may withdraw or take away your permission to use and disclose your health information at any time. You can do so by notifying Andrea Nugent, the study manager, in writing. Send your written withdrawal notice to:

Andrea Nugent
Ingenix
MN002-0258
12125 Technology Drive
Eden Prairie, MN 55344

If you withdraw your permission, you will not be able to continue being in this study. All information that has already been collected for study purposes will be sent to the study sponsor.

HEALTH INFORMATION RELEASE FORM

Authorization

I, ___________________________________ (print name), give UnitedHealthcare and its subsidiaries/affiliates, and their laboratories, providers, pharmacies and other service providers, permission to use and disclose my individually identifiable health information that may be contained in enrollment, medical and pharmacy claims information, lab results and benefit records to Ingenix, MN002-0258, 12125 Technology Drive, Eden Prairie, MN 55344 for the purposes described below.

1. Ingenix may:
   - use and share this health information to conduct the research in connection with the study, "Factors Affecting Administration and Completion of Human Papillomavirus (HPV) Vaccine Regimens";
   - disclose this health information as required by law and to representatives of government agencies, review boards, my health care providers and other persons who are required to watch over the safety, effectiveness, and conduct of research; and
   - remove from this health information my name and other information that could be used to identify me.
2. Once information that could be used to identify me has been removed, the information that remains is no longer subject to this Authorization and may be used and disclosed by Ingenix as permitted by law, including for other research purposes.
3. Once my health information has been disclosed to a third party, federal privacy laws may no longer protect it from further disclosure. However, Ingenix agrees to protect this health information by using and disclosing it only as permitted by me in this Authorization. Also, no publication about the research will reveal my identity without my specific written permission. These limitations continue even if I revoke (take back) this Authorization.
4. I have read that:
   - I do not have to sign this Authorization, but if I do not, I will not be allowed to participate in the research. If I do not participate, my medical care will not be affected and I will not lose any benefits to which I am entitled.
   - I may change my mind and revoke (take back) this Authorization at any time. To revoke this Authorization, write to: Andrea Nugent, Ingenix, MN002-0258, 12125 Technology Drive, Eden Prairie, MN 55344. If I revoke this Authorization, the information already obtained by Ingenix may remain a part of the research.
   - While the research is in progress, I will not be allowed to look at or copy my health information until after the research is completed.
5. This Authorization does not have an expiration (ending) date.
6. I will receive a copy of this Authorization after I have signed it.

____

Signature of Subject **Date**

____

Printed Name of Subject

SURVEY INSTRUCTIONS

This survey will ask you a series of questions about women's health issues, vaccinations, and awareness of cervical cancer and how to help prevent this disease. It should take approximately 15-20 minutes to complete. For each question, please read the instructions and use a pen or pencil to mark the box with an "X" that best corresponds to your answer.

For some questions, you may be asked to skip to another question based on the answer you choose. When this happens, you will see an arrow that tells you what question to answer next. Please follow these instructions carefully.

SECTION 1: ATTITUDES ABOUT YOUR HEALTH

1. First, we want to ask you about your health. Below is a list of statements which may or may not describe your attitudes about your health. Please read each statement and indicate how much you agree or disagree with it by marking the appropriate box with an "X".

	Strongly Disagree	Moderately Disagree	Slightly Disagree	Slightly Agree	Moderately Agree	Strongly Agree
a. If I get sick, it is my own behavior which determines how soon I get well again.	1☐	2☐	3☐	4☐	5☐	6☐
b. No matter what I do, if I am going to get sick, I will get sick.	1☐	2☐	3☐	4☐	5☐	6☐
c. Having regular contact with my physician is the best way for me to avoid illness.	1☐	2☐	3☐	4☐	5☐	6☐
d. Most things that affect my health happen to me by accident.	1☐	2☐	3☐	4☐	5☐	6☐
e. Whenever I don't feel well, I should consult a medically trained professional.	1☐	2☐	3☐	4☐	5☐	6☐
f. I am in control of my health.	1☐	2☐	3☐	4☐	5☐	6☐
g. My family has a lot to do with my becoming sick or staying healthy.	1☐	2☐	3☐	4☐	5☐	6☐
h. When I get sick, I am to blame.	1☐	2☐	3☐	4☐	5☐	6☐
i. Luck plays a big part in determining how soon I will recover from an illness.	1☐	2☐	3☐	4☐	5☐	6☐
j. Health professionals control my health.	1☐	2☐	3☐	4☐	5☐	6☐
k. My good health is largely a matter of good fortune.	1☐	2☐	3☐	4☐	5☐	6☐
l. The main thing which affects my health is what I myself do.	1☐	2☐	3☐	4☐	5☐	6☐
m. If I take care of myself, I can avoid illness.	1☐	2☐	3☐	4☐	5☐	6☐

	Strongly Disagree	Moderately Disagree	Slightly Disagree	Slightly Agree	Moderately Agree	Strongly Agree
n. Whenever I recover from an illness, it's usually because other people (for example, doctors, nurses, family, friends) have been taking good care of me.	1☐	2☐	3☐	4☐	5☐	6☐
o. No matter what I do, I'm likely to get sick.	1☐	2☐	3☐	4☐	5☐	6☐
p. If it's meant to be, I will stay healthy.	1☐	2☐	3☐	4☐	5☐	6☐
q. If I take the right actions, I can stay healthy.	1☐	2☐	3☐	4☐	5☐	6☐
r. Regarding my health, I can only do what my doctor tells me to do.	1☐	2☐	3☐	4☐	5☐	6☐

SECTION 2: ATTITUDES ABOUT VACCINES

2. These next questions ask about your opinions regarding vaccines (shots to help prevent disease). Please read each statement and indicate how much you agree or disagree with it by checking the appropriate box.

	Strongly Disagree	Moderately Disagree	Slightly Disagree	Slightly Agree	Moderately Agree	Strongly Agree
a. Doctors give out too many vaccines.	1☐	2☐	3☐	4☐	5☐	6☐
b. Vaccines are a good way to protect public health.	1☐	2☐	3☐	4☐	5☐	6☐
c. I do not like the idea of vaccines.	1☐	2☐	3☐	4☐	5☐	6☐
d. Shots are very painful.	1☐	2☐	3☐	4☐	5☐	6☐
e. Vaccines are generally safe.	1☐	2☐	3☐	4☐	5☐	6☐
f. Getting vaccinated can make you sick.	1☐	2☐	3☐	4☐	5☐	6☐
g. Vaccines are dangerous.	1☐	2☐	3☐	4☐	5☐	6☐
h. I am not afraid of shots.	1☐	2☐	3☐	4☐	5☐	6☐
i. Vaccines are a way to take good care of myself now and in the future.	1☐	2☐	3☐	4☐	5☐	6☐
j. Needles do not bother me at all.	1☐	2☐	3☐	4☐	5☐	6☐
k. Vaccines are effective.	1☐	2☐	3☐	4☐	5☐	6☐

SECTION 3: ABOUT YOU

The next questions ask you some general questions about yourself. Your answers will help us describe the types of women participating in this study. Please read each question carefully and respond by marking the appropriate boxes with an "X".

3. How would you describe your overall health? *(Please select one answer only)*

1☐ Poor
2☐ Fair
3☐ Good
4☐ Very Good
5☐ Excellent

4. In what year were you born? Please enter as a four-digit number (e.g., 1983).

Year of birth: [1][9][ ][ ]

5. Which of the following do you consider yourself to be? *(Check all that apply)*

1 ☐ American Indian or Alaska Native
2 ☐ Asian
3 ☐ Black or African American
4 ☐ Hispanic or Latino
5 ☐ Native Hawaiian or Other Pacific Islander
6 ☐ White
7 ☐ Other: ____________________

6. What is the highest level of education you have completed or the highest degree you have received?

1 ☐ Less than high school
2 ☐ Some high school
3 ☐ High school or equivalent (e.g., GED)
4 ☐ Some college, but no degree
5 ☐ Two-year degree (community or technical)
6 ☐ College graduate
7 ☐ Graduate school

7. What is your marital status?

1 ☐ Single, never married
2 ☐ Married
3 ☐ Divorced
4 ☐ Separated
5 ☐ Widowed

8. Which of the following best describes your living situation? *(Check all that apply)*

1 ☐ I live with one or both of my parents
2 ☐ I live away at school most of the year
3 ☐ I live alone
4 ☐ I live with my partner/husband/fiancé
5 ☐ I live with other relatives
6 ☐ I live with my child(ren)
7 ☐ I live with a roommate (someone who is not a relative)

9. Which one of the following best describes your employment status?

1 ☐ Employed full-time
2 ☐ Employed part-time
3 ☐ Self-employed
4 ☐ Not employed, but looking for work
5 ☐ Not employed and not looking for work
6 ☐ Retired
7 ☐ Student
8 ☐ Homemaker

SECTION 4: ATTITUDES ABOUT REPRODUCTIVE HEALTH

This section of the survey consists of a list of statements, which may or may not describe your attitudes about reproductive health, including sexual health. Some statements may contain descriptions of medical tests that are unfamiliar to you. Please use these definitions below to help you answer the questions in this section.

- **Pap test/Pap smear:** A procedure in which cells are scraped from the cervix for examination under a microscope. It is used to detect cancer and changes that may lead to cancer. A Pap smear can also show noncancerous conditions, such as infection or inflammation.
- **Gynecological/pelvic examination:** A physical examination in which the health care professional will feel for lumps or changes in the shape of the vagina, cervix, uterus, fallopian tubes, ovaries, and rectum. The health care professional will also use a speculum to open the vagina to look at the cervix and take samples for a Pap test. This may also be called an internal exam.

10. Please read each statement and indicate how much you agree or disagree with it by marking the appropriate box with an "X". If you are uncertain about how to answer a specific question, please just choose the response that comes closest to your opinion. If a particular question does not apply to you, please write "NA" (not applicable) next to the question.

	Strongly Disagree	Moderately Disagree	Slightly Disagree	Slightly Agree	Moderately Agree	Strongly Agree
a. I am comfortable discussing sexual health issues with a doctor or nurse.	1☐	2☐	3☐	4☐	5☐	6☐
b. I am comfortable discussing sexual health issues with others such as family or friends.	1☐	2☐	3☐	4☐	5☐	6☐
c. I get a gynecological or pelvic exam according to my doctor's/health care provider's advice.	1☐	2☐	3☐	4☐	5☐	6☐
d. I don't mind getting a gynecological/pelvic exam.	1☐	2☐	3☐	4☐	5☐	6☐
e. Gynecological/pelvic exams are necessary to stay healthy.	1☐	2☐	3☐	4☐	5☐	6☐
f. I get a Pap test/Pap smear according to my doctor's/health care provider's advice.	1☐	2☐	3☐	4☐	5☐	6☐
g. It is very important to have an annual pelvic exam.	1☐	2☐	3☐	4☐	5☐	6☐
h. Cervical cancer is a devastating disease.	1☐	2☐	3☐	4☐	5☐	6☐
i. Genital warts are an embarrassing condition.	1☐	2☐	3☐	4☐	5☐	6☐

SECTION 5: AWARNESS OF CERVICAL CANCER VACCINE

Next you will be asked a series of questions about vaccinations to help protect against cervical cancer and human papillomavirus (HPV) infection. Please read each question carefully and respond by marking the appropriate box with an "X".

11. Have you ever heard of a vaccine to help prevent cervical cancer or HPV (e.g., Gardasil® or Cervarix®)?

1☐ Yes ➔ *Go to Question 12*
2☐ No ➔ **STOP - You have reached the end of the survey. Please return this completed booklet using the enclosed postage-paid envelope. Thank you for your participation.**

12. If you answered "Yes" to Question 11 about having heard of Gardasil® or Cervarix®, how important do you think the vaccine to help prevent cervical cancer is for you?

1☐ Not at all important
2☐ Not very important
3☐ Somewhat important
4☐ Very important

13. Have you discussed the vaccine to help prevent cervical cancer with a doctor?

1☐ Yes ➔ *Go to Question 14*
2☐ No ➔ Skip to SECTION 6: FUTURE CERVICAL CANCER VACCINE USE on page 13

14. Did a doctor recommend that you get the vaccine to help prevent cervical cancer?

1☐ Yes ➔ *Go to Question 15*
2☐ No ➔ Skip to SECTION 6: FUTURE CERVICAL CANCER VACCINE USE on page 13

15. If you answered "Yes" to Question 14 about a doctor recommending the cervical cancer vaccine, how <u>strongly</u> did the doctor recommend you get the cervical cancer vaccine on a scale from 1 to 5?

1☐ **A doctor did not** strongly recommend the vaccine
2☐
3☐
4☐
5☐ **A doctor strongly** recommended the vacine

16. Have you received one or more doses of the vaccine to help prevent cervical cancer?

1☐ Yes ➔ *Go to Question 17*
2☐ No ➔ Skip to SECTION 6: FUTURE CERVICAL CANCER VACCINE USE on page 13

17. Which of the following best describes where you received the cervical cancer vaccine(s)? *(Check all that apply)*

1☐ Primary care provider/Family physician office
2☐ College health center
3☐ Pharmacy
4☐ Public health clinic/Community health clinic
5☐ Planned Parenthood/Family planning clinic
6☐ Obstetrician/gynecological (OB/GYN) office
7☐ Pediatrician office
8☐ Other (describe): ______________________________

18. The full course of the vaccine to help prevent cervical cancer requires three doses in total. How many doses of the cervical cancer vaccine have you received to date?

1☐ One dose
2☐ Two doses
3☐ Three doses

➔ **If you answered three doses, STOP - You have reached the end of the survey. Please return this completed booklet using the enclosed postage-paid envelope. Thank you for your participation.**

19. If you have not received all three doses yet, do you intend to do so?

1☐ Yes ➔ *Skip to Question 20*
2☐ No ➔ *Go to Question 19a*

19a. Why do you not intend to receive all three doses? ***(Check all that apply)***

1☐ Cost of the vaccine
2☐ I had a reaction to the vaccine
3☐ I found the injection to be too painful
4☐ Vaccine was inconvenient to get
5☐ I worry about the vaccine's safety
6☐ I got pregnant
7☐ I got married
8☐ I am no longer at risk of human papillomavirus (HPV) infection
9☐ Other (describe): ________________

If you answered Question 19a, then STOP, you have reached the end of the survey. Please return this completed booklet using the enclosed postage-paid envelope. Thank you for your participation.

20. The full course of the vaccine to prevent cervical cancer vaccine requires three doses in total. How will you remember to receive your next future dose? ***(Check all that apply)***

1☐ An appointment has been scheduled to get the next dose
2☐ When it is time for the next dose, I will set up an appointment
3☐ I will wait for the doctor to bring it up
4☐ Other (describe): ________________

21. Which of the following, if any, does/did your doctor do to help you remember when it is/was time for the next administration of the cervical cancer vaccine? ***(Check all that apply)***

1☐ Gives reminder cards when first dose was received
2☐ Sends an e-mail reminding me to get my next dose
3☐ Sends a postcard in the mail to remind me of my next dose
4☐ Asks me to make an appointment before leaving the doctor's office
5☐ Sends a phone call reminder
6☐ None of the above
7☐ I do not know
8☐ Other (describe): ________________

If you answered Question 21, the STOP, you have reached the end of the survey. Please return this completed booklet using the enclosed postage-paid envelope. Thank you for your participation.

SECTION 6: FUTURE CERVICAL CANCER VACCINE USE

Please answer these final questions only if you have never received a dose of cervical cancer vaccine. Read each question carefully and mark the appropriate answer with an "X".

22. If you have not received a single dose of the cervical cancer vaccine, in the future how likely are you to:

	Not at all likely	Not very likely	Somewhat likely	Very likely	Extremely likely
a. Ask a doctor to get this vaccine?	1☐	2☐	3☐	4☐	5☐
b. Do additional research on this vaccine?	1☐	2☐	3☐	4☐	5☐
c. Discuss the vaccine with a doctor?	1☐	2☐	3☐	4☐	5☐
d. Make an appointment to get the vaccine?	1☐	2☐	3☐	4☐	5☐
e. Do nothing to get the vaccine?	1☐	2☐	3☐	4☐	5☐

23. If you answered that you are somewhat likely, very likely or extremely likely to "Do nothing to get the vaccine" in Question 22e above, please indicate the reason(s) why you would not do anything. *(Check all that apply)*

1☐ I am not sexually active
2☐ The vaccine is too new
3☐ I am married or in a exclusive (monogamous) relationship
4☐ I am unsure if my insurance would cover the vaccine cost
5☐ I do not have enough information about this vaccine
6☐ I am concerned about the side effects
7☐ I am pregnant or trying to conceive
8☐ My doctor recommended against getting the vaccine
9☐ I cannot afford the cost of the vaccine
10☐ Other (describe): ______________________

THANK YOU! You have reached the end of the survey.
Please review your responses to make sure all questions have been answered,
check to make sure you have signed pages 4 and 6,
and mail the survey in the postage-paid envelope.
